# Development of a Perinatal Palliative Care Model at a Level II Perinatal Center Supported by a Pediatric Palliative Care Network

**DOI:** 10.3389/fped.2020.574397

**Published:** 2021-01-15

**Authors:** Marco Bolognani, Paola Daniela Morelli, Isabella Scolari, Cristina Dolci, Valentina Fiorito, Francesca Uez, Silvia Graziani, Barbara Stefani, Francesca Zeni, Gino Gobber, Elena Bravi, Saverio Tateo, Massimo Soffiati

**Affiliations:** ^1^Department of Neonatology, Santa Chiara Hospital, Trento, Italy; ^2^Azienda Provinciale per i Servizi Sanitari (APSS), Trento, Italy

**Keywords:** life limiting conditions, pediatric palliative care, perinatal palliative care, life threatening conditions, fetal diagnosis

## Abstract

**Objective:** To describe the model build up to take care of fetuses and newborns eligible to perinatal palliative care (PnPC) followed in an Italian II level perinatal center.

**Methods:** Retrospective chart review of all fetuses and newborn infants eligible to PnPC admitted to level II perinatal center within a 4 years period.

**Results:** Forty-five of 848 infants (0.5%) referred to II level NICU were eligible to PnPC. Twenty-seven percentage had fetal diagnosis. Twenty percentage were preterm infants at the limit of viability, 35% were newborns with life limiting or life threatening disease diagnosed *in utero* or at the postnatal ward, 45% were newborns not responding to intensive care intervention with high health care needs or medical complexity. Fifty-seven percentage of neonates admitted to NICU died before discharge, while 16 (35% of population considered) were discharged home. Median age at death was 4 days after birth, and delivery room death immediately after birth occurred in six patients (13%).

**Conclusions:** Despite the paucity of our population and the high variability in disease trajectories the perinatal palliative care program build up in our region provides a reproducible method for a structured taking in charge of fetuses and neonates eligible to PnPC and their families, from the time of diagnosis to bereavement, in both outpatient and inpatient settings.

## Introduction

Perinatal palliative care (PnPC) focuses on coordinated care strategies for babies born at the edge of viability, fetuses and neonates with confirmed or potential diagnosis of life-limiting and life-threatening conditions, neonates who become critically ill during a neonatal intensive care unit (NICU) stay and are not responding to aggressive medical management (1). PnPC implies multidisciplinary, global and continuous care of the family from the time of diagnosis to the end-of-life. Initial combination of life-prolonging and disease-modifying treatment with palliative care could afterwards turn to exclusive attention to quality of life and comfort care because of inappropriateness and futility of previous treatments (2).

Lack of evidence-based empirical studies to identify the best model for PnPC (2) and the insufficient diffusion of structured PnPC programs leaves many aspects challenging in an otherwise growing field of evidence. A recent national survey reports that only 30% of Italian NICUs offer a structured PnPC program (3) despite a national law of 2010 which protects the citizen's right to access palliative care (4).

The pediatric palliative care (PPC) Network in our region, since 2016 has started to develop a program for PnPC in collaboration with level II perinatal center, considering national and international suggestions and experiences (5–10). Palliative care is disposable at any time in the pregnancy cycle: when the fetus is *in utero*, when the pregnancy is ended, when there is an early induction, after a live birth and transition to palliative care in the NICU or upon discharge home (5). PnPC planning and care is provided at the time of diagnoses on through the whole trajectory of life, and is applied indifferently in hospital settings and at home. PPC team coordinates care with hospital and local staff in a collaborative, community-based and family centered frame.

## Objective

The aim of this study was to describe the model build up to take care of fetuses and newborns eligible to perinatal palliative care and followed in an Italian II level perinatal center.

## Methods

The study population consisted of all fetuses and neonates eligible to PnPC who were cared for in the Trentino II level perinatal center from January 1, 2016 to May 31, 2020. All eligible participants were identified by examining retrospectively hospital documentation and were subsequently distributed in three groups according to current literature about eligibility to PnPC (1, 10, 11).

Group 1: newborns at the threshold of viability (birth weight <500 g or gestational age under 24 weeks).

Group 2: newborns with life limiting or life threatening disease diagnosed *in utero* or at the postnatal ward.

Group 3: newborns not responding to intensive care intervention with high health care needs or medical complexity.

### PnPC Model Description

**Figure 1:**
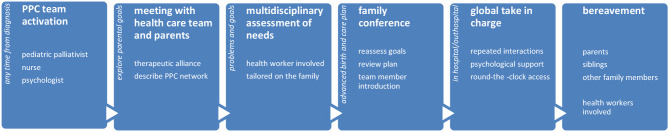
PnPC model description.

The PPC team can be activated at any time from diagnosis of a fetal or neonatal condition eligible to palliative care (Figure 1). This first contact serves to meet the parents, describe PPC network, establish a therapeutic alliance and focus on exploring parental goals.

Thereafter a multidisciplinary assessment of problems and needs takes place involving: parent(s) and members of their support system, obstetrician, neonatologist, PPC team (pediatrician, nurse, and psychologist), other specialists (neuropsychiatrist, pediatric cardiologist, and geneticist) if appropriate. Problems, needs and goals are reassessed through a model utilized in PPC (12), exploring also specific perinatal needs and tailored on each family, considering every available resource in order to achieve the endorsed goals.

The PPC team elaborates subsequently an individualized advanced birth and care plan which contains anticipatory management (Table 1).

**Table 1:** Content of advanced birth and care plan.

Birth care plan	• Place • Timing • Mode • Monitoring in labour • People to be present at delivery • Care provided at delivery • Which (if any) diagnostic interventions to be done • Postnatal care • Skin to skin
Palliative care plan	• Pain • Medical system access and quality • Family oriented care • Dignity and respect • Decision making • Psychosocial • Spiritual symptoms treatment plan • Family support (siblings and grandparents)
Bereavement care plan	• Arranging for spiritual/cultural care • Psychosocial support • Supporting memories

A family conference is than arranged to review the individualized birth and care plan and introduce PPC network team members. Parents are given the opportunity to re-express their goals of care and they are told that the plan will be continuously reassessed. These meetings range in length from 1 to 3 h.

The plan is finally discussed with all the health care providers involved and targeted training on PPC is offered and provided to all the nodes of the network involved (obstetrician, neonatologist, nurses, psychologist, emergency services, social service, and home care service).

During prenatal, birth and postnatal periods the PPC team has repeated interactions with parents and the health care providers involved. Psychological support is provided for parents, siblings and other family members and the team caring for the fetus or neonate. This support proved to be particularly valuable in providing age-appropriate, honest and concrete explanations to young children to help them understand.

At time of delivery one of the members of the PPC team is present or available for consultation.

After delivery for neonates who survive the immediate newborn period rooming-in–if appropriate and desired–is ensured. Infants who survive long enough to be discharged from the hospital are taken care of at home, were planned physician visits take place once a fortnight and planned nurse visits twice a week. Visits of psychologists, social workers, physiotherapists are planned according to individual needs of each family. Patients and parents have a round-the-clock access to telephone consultation service, and intervention visits are a standard procedure in case of a patient's health deterioration. PPC team takes care of bereavement counseling and provides support for health workers involved who are dealing with emotional distress. PPC is free of charge and all medical equipment is loaned to the families free of charge.

## Results

Between January 2016 and May 2020, 9.849 neonates were born at the Trentino II level perinatal center “S Chiara” and 848 neonates were cared for at the II level NICU. The center counts an annual working volume of 2,200 prenatal diagnostic ultrasound investigations, 1,400 screening techniques for chromosomal abnormalities, 80 villocentesis and 40 amniocentesis. During the period in exam 62 voluntary interruptions of pregnancy, 60 spontaneous abortions and 15 fetal deaths took place.

**Figure 2:**
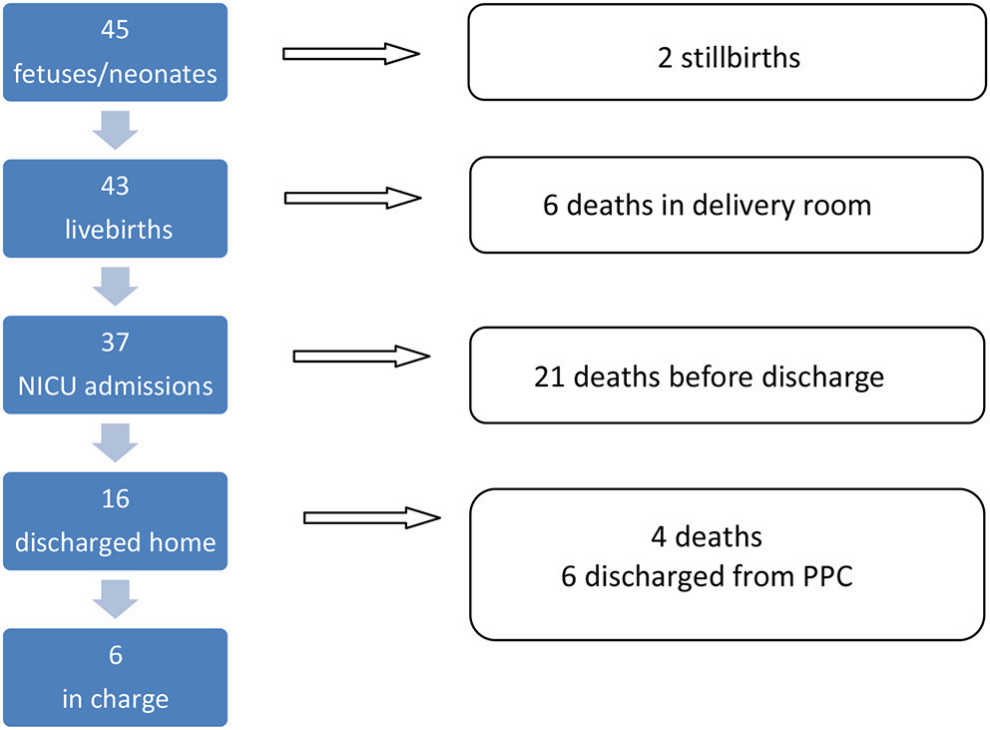
Outcome of 45 neonates.

Forty-five neonates (0.5% of all NICU admissions and 0.05% of all newborns) eligible to PnPC were retrospectively identified, 12 (27%) of them had fetal diagnoses, two stillbirths and 43 livebirths. Median (range) gestation at delivery was 31 (23–41) weeks. Babies survived for 1 h to 565 days (median 4 days). Deaths in the delivery room shortly after birth accounted for 13%. Twenty one out of 37 neonates (57%) admitted to the NICU died before discharge (median age at death was 4 days). Of the 21 patients who died, 12 (57%) died while receiving maximal support measures (including cardiopulmonary resuscitation), and 9 (44%) died under primary comfort care or after withholding or withdrawal of life support measures. 16 (35%) were discharged home. Outcome is reassumed in Figure 2 and main characteristics of the 45 fetuses and neonates analyzed in the study are summarized in Table 2.

**Table 2:** Distribution of neonates eligible to PnPC.

**Group**	**1**	**2**	**3**
	**Newborns at the threshold of viability (birth weight <500 g or gestational age under 24 weeks)**	**Newborns with life limiting or life threatening disease diagnosed *in utero* or at the postnatal ward**	**Newborns not responding to intensive care intervention with high health care needs or medical complexity**
nr (%)	9 (20)	16 (35)	20 (45)
Died before discharge			
nr (%)	9 (100)	8 (50)	12 (60)
Age at death	^*^4.5 ± 4.6 (3) 0.04–13 days	^*^0.8 ± 1.5 (4) 0.04–4 days	^*^5 ± 7 (2.5) 0.4–21 days
In charge of PPC			
nr (%)	1 (11)	13 (80)	16 (80)
Duration	1day	^*^257 ± 205 (316) 18–494 days	^*^163 ± 240 (47) 1–853 days
After discharge			
Died at home	0	0	0
Died in hospital	0	4	0
Discharged from PPC	0	0	6
In charge to PPC	0	4	2
	BW 490 (415–522)	Trisomy 13, 18 *n* = 4	HIE *n* = 8
	GA 23 (23–25)	Thanatophoric dwarfism *n* = 1	Severe brain injury *n* = 3
		Potter's syndrome *n* = 2	MOF *n* = 2
		Metabolic disease *n* = 2	Dysphagia and CLD *n* = 7
		Multiple malformations *n* = 4	
		Epidermolysis bullosa *n* = 1	
		Cardiomyopathies *n* = 2	

*BW, birth weight; GA, gestational age; HIE, hypossic-ischemic encephalopathy; MOF, multiorgan failure; CLD, chronic lung disease*.

* *Mean ± standard deviation (median) min-max*.

In group 1, two out of nine newborns died in delivery room, while seven died in intensive care unit receiving support measures (median age at death was 3 days). Only in one case PPC team has been involved.

In group 2, besides two stillborns, four out of 16 newborns died in delivery room, two in NICU and eight were discharged home. Four of these eight infants died in hospital (place of death chosen by the parents) receiving comfort care at a median age at death of 111 days. Four to date are in charge of CPP network.

Group 3 includes 20 neonates, 12 of them died in NICU and eight were discharged home and taken care of by the PPC network. For six of these it has been possible to discharge them from PPC network and entrust them to the family pediatrician because of the improvement of clinical conditions and the reduction of complexity of care. Two to date are in charge of CPP network.

Altogether for 30 of the 45 fetuses and newborns eligible to PPC (67%) PPC team has been involved. The involvement grew during the period analyzed (from 50% in 2016 to 88% in 2019) and differed between group 1 and 2–3, respectively (11 vs. 80%).

The duration of PPC among all fetuses/neonates varied from 1 to 853 days with a median duration of 99 days. For those discharged home instead from 18 to 853 days with a median duration of 316 days. In this group of 16 infants the most frequent clinical problems included neurological symptoms (69%) and dysphagia (81%). A high percentage of the infants (62%) had a feeding tube (naso-orogastric) inserted, 20% percutaneous endoscopic gastrostomy and 12% tracheostomy, 40% of patients needed mechanical ventilation at discharge one patient had a central venous catheter inserted. Fifteen of all the 30 families taken in charge agreed and received permanent psychological care and every family received social supports needed.

Eight of the 45 pathways begun at prenatal diagnosis of a life limiting or life threatening condition, three out of eight were achieved beyond 20 weeks of gestational age (13), whereas the other five diagnoses were obtained within 22 weeks of gestation but parents decided to continue pregnancy.

## Discussion

This study retrospectively analyzes data of all fetuses and neonates cared for in the II level perinatal center of Trento during a 4 year period from January 2016 to May 2020. Regional PPC network began to elaborate a PnPC program since 2016.

In our experience newborns belonging to group 1 rarely were notified to PPC team thus reproducing results of other experiences (11). Reasons for this may be the difficulty in acknowledging prognosis in this subgroup of patients and the reduced exposure of treating team to this kind of patient (one every 5–6 months), moreover there is to consider lack of palliative care education in medical and nursing school in our country.

Whereas, the involvement of the PPC network is growing in regard of fetuses and infants of group 2 because health professionals were previously unfamiliar with alternative management such as PnPC and the growing evidence of the importance of early referral to PnPC has been shared and recognized. Even if lethal fetal abnormalities are uncommon and most couple request termination after a diagnosis of lethal abnormality, late diagnoses will continue to arise as incidental discoveries and many of these babies will be life born with a variable and unpredictable life course (14) thus requiring adequate and comprehensive care.

Outcome of group 3 results very heterogeneous but takes in account a group of patients which can take advantage of an early referral to PnPC because of the clinical complexity of their condition. Health care providers are faced with the difficult task to revise treatment from aggressive cure-oriented intervention to palliative care goals coping with personal values and feelings.

Since the implementation of the model described, an improvement has been noted by health workers involved in terms of facilitated decision making and consensus building, improvement of therapeutic alliance, better flow of information in a framework characterized by difficult and complex matters and many “gray zones.”

Taking inspiration from this first years of networking a formal education plan for possible staff members involved is in a planning stage and team support in order to manage properly communication management and emotional containment has been completed (15, 16).

NICU may not be the optimal place for provision of palliative care, but according to the evidence that location is not as important as the “mind set” of persons involved (1) and considering our experience appropriate levels of PnPC can be accomplished through attitudes, appropriate skills training and shared birth and advanced care plans.

## Conclusions

At a first glance our data depict the broad variability health workers have to face in managing patients and families in need of PnPC. The vast clinical conditions with different and unpredictable trajectories of life in terms of length and burden of care requires multidisciplinarity, communication between services, forecasting of problems, flexibility and adaptation to different settings. Despite our small population and the high variability in disease trajectories the perinatal palliative care program developed in our region provides a replicable method for a structured responsibility of fetuses and neonates eligible to PnPC and their families, from the time of diagnosis to bereavement, in both outpatient and inpatient settings. Outcomes from our program support development of similar programs to meet the needs of the families who do not wish to terminate pregnancies or who do not wish to use intensive care services for babies, if born alive and as near to their home as possible.

## Data Availability Statement

The raw data supporting the conclusions of this article will be made available by the authors, without undue reservation.

## Ethics Statement

Ethical review and approval was not required for the study on human participants in accordance with the local legislation and institutional requirements. Written informed consent from the participants' legal guardian/next of kin was not required to participate in this study in accordance with the national legislation and the institutional requirements.

## Author Contributions

MB, PM, and IS contributed in collecting data. CD, VF, and FU contributed in building the PnPC program. SG, BS, and FZ participated in defining the pathways. EB, GG, ST, and MS participated in manuscript review. All authors contributed to the article and approved the submitted version.

## Conflict of Interest

The authors declare that the research was conducted in the absence of any commercial or financial relationships that could be construed as a potential conflict of interest.
